# A novel link between keratoderma and cardiomyopathy: contiguous gene deletion involving the desmoglein gene cluster

**DOI:** 10.1111/bjd.15584

**Published:** 2017-11-16

**Authors:** S. Brown, J. Ahmed, S. Zwolinski, P. Brennan, N. Rajan

**Affiliations:** ^1^ Institute of Genetic Medicine Centre for Life Newcastle upon Tyne NE1 3BZ U.K.; ^2^ Cardiothoracic Services The Newcastle upon Tyne Hospitals NHS Foundation Trust Freeman Hospital Freeman Road, High Heaton Newcastle upon Tyne NE7 7DN U.K.

dear editor, Desmocollin and desmoglein are important proteins in intercellular adhesion in both the skin and the heart.^1^ Heterozygous mutations in the desmocollin 2 (*DSC2*)^2^ and desmoglein 2 (*DSG2*)^3^ genes can both result in desmosomal dysfunction in cardiomyocytes, leading to development of fibrofatty tissue, termed arrhythmogenic right ventricular cardiomyopathy (ARVC). Heterozygous truncating mutations in desmoglein 1 (*DSG1*), an intervening gene, can present as a striate palmoplantar keratoderma, while homozygous *DSG1* mutations can cause severe dermatitis, allergies and metabolic wasting.^4^ Unlike mutations in plakoglobins and desmoplakins, which affect both tissues, the desmogleins, desmocollins and plakophilins typically affect either the skin or the heart.^1^ Here we present a patient with a novel heterozygous deletion resulting in loss of a 2·6‐Mb region including *DSC2*,* DSG2* and *DSG1*, associated with ARVC and keratoderma (Fig. 1).

**Figure 1:**
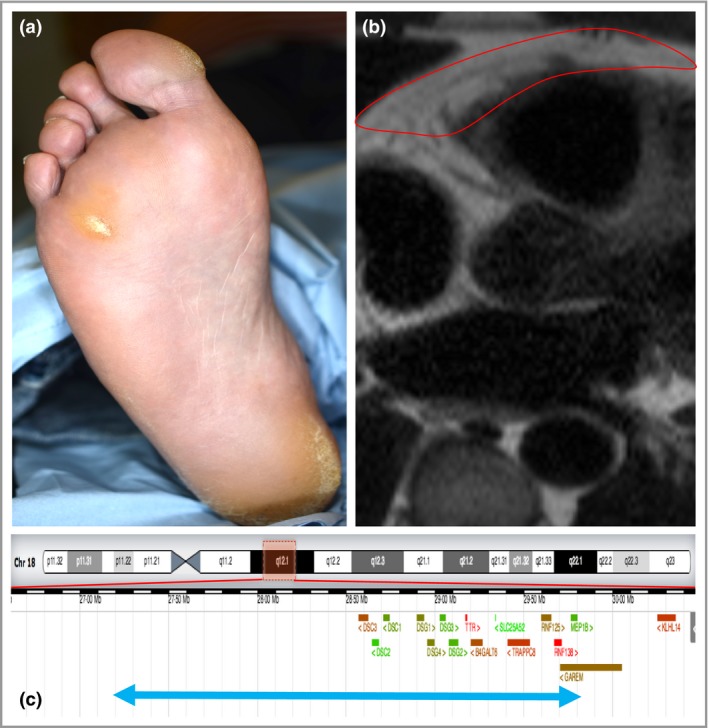
(a) Focal keratoderma predominantly over pressure areas and regions in contact with footwear. (b) Cardiac magnetic resonance imaging with the right ventrical outlined in red, where fibrofatty change is noted as a high‐signal region. (c) Span of the 2·6‐Mb deletion indicated by the blue arrow, with genes indicated in red and green.

A 52‐year‐old man with congenital midpenile shaft hypospadias presented for correction of a urological stricture. He had focal areas of thickening of the skin of the soles since his second decade that were exacerbated during military training. His family history was positive for ischaemic heart disease in three maternal uncles and a brother, requiring a permanent pacemaker, but none had keratoderma. His father had died suddenly in his sixth decade. Clinical examination revealed focal palmoplantar keratoderma on the soles of the feet and the base of his fingers. There were no hair or nail changes.

Preoperative cardiac investigations included an electrocardiogram, which showed T‐wave inversion in lead V3 and Q waves in leads II, V5 and V6. This abnormality prompted further investigations, of which cardiac magnetic resonance imaging demonstrated evidence of right ventricular dysfunction and fibrofatty changes consistent with a diagnosis of ARVC (Fig. 1b). Genetic sequence analysis was performed, which was normal in genes associated with ARVC (*DES*,* DSC2*,* DSG2*,* DSP*,* JUP*,* LMNA*,* PKP2* and *TMEM43*). Multiplex ligation‐dependent probe amplification (MLPA) was performed, which suggested a *DSC2* and *DSG2* heterozygous deletion. Array comparative genomic hybridization (aCGH) was then performed, which revealed a heterozygous deletion spanning 2·6 Mb, including *DSC2*,* DSG2* and 13 other genes: arr[GRCh37] 18q12·1(27188342_29793320)x1 (Table S1; see Supporting Information). Testing of three of his siblings, the patient's mother and one of his two daughters did not reveal this change.

We present a novel genetic mechanism linking keratoderma and cardiomyopathy by contiguous gene deletion involving genes that encode desmosomal proteins. This adds to known causes including Naxos disease (plakoglobin gene deletions)^5^ and Carvajal syndrome (recessive desmoplakin gene mutations),^6^ which are characterized by cardiomyopathy, palmoplantar keratoderma and woolly hair. ARVC can vary in severity, from being asymptomatic to causing ventricular arrhythmias and sudden death in the fourth decade. In our patient, the skin and heart phenotypes are both mild, and he has been managed conservatively with beta‐blockers. DNA sequencing of ARVC genes yielded a negative result, which prompted further investigation with genetic assays that gave information on gene dosage, namely MLPA and aCGH.

Our case highlights the need for alternative techniques to detect heterozygous deletions in patients presenting with keratoderma and cardiomyopathy. The region deleted in our patient (Case 303180) has been deleted on one allele in three other patients reported on DECIPHER,^7^ an online repository of matched genetic and clinical phenotypic data. In the other cases, the phenotypes reported are developmental delay, short stature and obesity (Case 286198; 12·7‐Mb deletion); malformation of the heart and great vessels, and intellectual disability (Case 260121; 13·4‐Mb deletion); and behavioural abnormalities and motor delay (Case 276030; 14·5‐Mb deletion). Notably, ARVC and keratoderma have not been reported in these individuals, suggesting that if the phenotype if present it is likely to be mild or less significant to the patient than the reported associations. The larger deletion in these patients includes more genes (up to 76 in total), making it difficult to discern the contributory impact of the deletion seen in our patient. Our findings suggest that gene dosage of this region is important in the maintenance of normal cardiac and cutaneous function, and highlight a novel link between cardiomyopathy and keratoderma.

## Supporting information


**Table S1** Genes within the 2·6‐Mb deleted region.
