# Supplementary material for: A novel link between keratoderma and cardiomyopathy: contiguous gene deletion involving the desmoglein gene cluster
Source: Br J Dermatol. 2017 Nov 16;178(1):284–5. doi: 10.1111/bjd.15584 (PMC5813196; doi:10.1111/bjd.15584)
Supplement: Supplementary file 1 — Table S1 Genes within the 2·6‐Mb deleted region. [file BJD-178-284-s001.docx]

**Table S1** Genes within the 2.6-Mb deleted region

| **Name** | **Location (chr)** | **Description** | **OMIM** | **Morbid** |
| --- | --- | --- | --- | --- |
| *DSC3* | 18 ^28569974^  _28622781_ | Desmocollin 3 | ✓ | ✓ |
| *DSC2* | 18 ^28645940^  _28682378_ | Desmocollin 2 | ✓ | ✓ |
| *DSC1* | 18 ^28709199^  _28742819_ | Desmocollin 1 | ✓ | - |
| *DSG1* | 18 ^28898052^  _28936992_ | Desmoglein 1 | ✓ | ✓ |
| *DSG4* | 18 ^28956740^  _28994875_ | Desmoglein 4 | ✓ | ✓ |
| *DSG3* | 18 ^29027758^  _29058665_ | Desmoglein 3 | ✓ | - |
| *DSG2* | 18 ^29078006^  _29128971_ | Desmoglein 2 | ✓ | ✓ |
| *TTR* | 18 ^29171689^  _29178974_ | Transthyretin | ✓ | ✓ |
| *B4GALT6* | 18 ^29202210^  _29265799_ | UDP-Gal:betaGlcNAc beta 1,4- galactosyltransferase, polypeptide 6 | ✓ | - |
| *SLC25A52* | 18 ^29339525^  _29340843_ | Solute carrier family 25, member 52 | ✓ | - |
| *TRAPPC8* | 18 ^29409136^  _29533099_ | Trafficking protein particle complex 8 | ✓ | - |
| *RNF125* | 18 ^29598335^  _29653176_ | Ring finger protein 125, E3 ubiquitin protein ligase | ✓ | ✓ |
| *RNF138* | 18 ^29671818^  _29711524_ | Ring finger protein 138, E3 ubiquitin protein ligase | ✓ | - |
| *GAREM* | 18 ^29704840^  _30050447_ | GRB2 associated regulator of MAPK1 | - | - |
| *MEP1B* | 18 ^29765032^  _29800367_ | Meprin A, beta | ✓ | - |
